# A method of rice panicle number counting based on improved CSRNet model

**DOI:** 10.3389/fpls.2025.1494564

**Published:** 2025-04-03

**Authors:** Junyan Zhu, Jiaxing Huang, Yiyuan Wang, Jingyu Wang, Yongxian Wen

**Affiliations:** ^1^ College of Agriculture, Fujian Agriculture and Forestry University, Fuzhou, Fujian, China; ^2^ College of Computer and Information Sciences, Fujian Agriculture and Forestry University, Fuzhou, Fujian, China; ^3^ Institute of Statistics and Application, Fujian Agriculture and Forestry University, Fuzhou, Fujian, China; ^4^ College of Computer and Data Science, Fuzhou University, Fuzhou, Fujian, China

**Keywords:** rice panicle counting, deep learning, CSRNet, yield estimation, automatic counting

## Abstract

**Introduction:**

Rice is one of the world's leading food crops, with nearly half of the world's population eating rice as their staple food. Rice yield is directly related to varieties, and the most intuitive agronomic trait of varietal yield is the number of grains per panicle.

**Methods:**

In this study, rice panicles are taken as the research object, and images of the panicles are captured using a smartphone. The CSRNet counting model based on deep learning is then improved and applied to the problem of counting the number of grains per panicle in rice.

**Results and discussion:**

The results show that the method of this study has a mean error value of 3.83% on the final validation set. On this basis, the development of rice per panicle counting APP based on Android terminal and batch counting software RiceGrainCounter based on PC terminal realizes real-time counting on Android terminal and batch counting on PC terminal, which can provide theoretical basis and technical support for rice per panicle counting.

## 1 Introduction

In crop breeding research, crop yield has always been a concern of agronomic researchers and how to improve crop yield remains a worldwide problem. In order to find the correlation between crop yield and variety, yield measurement is an essential part of the process. The yields of grain crops such as rice, wheat and maize are mainly closely related to the number of panicles per unit area, thousand-panicles quality and the grain number of per panicle. The traditional measurement of counting mainly relies on manual counting, which is time-consuming, inefficient and prone to errors, and the counting of thousand-panicles quality and the number of per panicle is destructive to crop panicles, which is not conducive to the reuse of materials (Tester and Langridge, 2010; Al-Tam et al., 2013). With the development of computer image technology, it is an inevitable trend to take pictures of grain crops such as rice, wheat, and corn, analyze the resulting images, and complete yield forecasts instead of manual measurements. Especially in recent years, research on crop yield prediction by incorporating deep learning has also made significant progress. For example, deep learning is utilized to count the number of panicles per unit area of wheat, rice, etc. and thus predict the yield in the field (Lu et al., 2017; Hasan et al., 2019; Bao et al., 2020; Yang et al., 2022; Yang, 2020). The number of panicles counted per unit area is often analyzed from the perspective of environmental impact on yield. However, the number of grains per panicle in grain crops such as rice, wheat, and corn are also an important agronomic trait that directly affects their yields (García et al., 2016; Li et al., 2004; Slafer et al., 2014; Xing and Zhang, 2010). Rapid and accurate measurement of the number of grains per panicle can improve the efficiency of scientific research and variety development. Therefore, counting the number of grains per panicle is a very important task.

The method of yield estimation using computer image technology provides a new approach to the problem of counting the number of grains per panicle in rice. There are two main traditional methods for measuring the number of grains per panicle using image technology: one involves counting the grains by extracting the outline of the panicle (Bleau and Leon, 2000; Crowell et al., 2014; Lin et al., 2014; Mandal, 2018), and the other is to count the grains by extracting the morphological features of the rice panicle (Al-Tam et al., 2013; Crowell et al., 2014; Ikeda et al., 2010). In recent years, an increasing number of researchers have applied deep learning to the count of grain number per panicle in rice. Wu et al. used image processing and deep learning algorithms (Faster RCNN+ResNet101 network) to detect the number of grains per spike of rice from rice spike images. They first extracted the features and generated the feature maps using ResNet101, and then used Faster RCNN for prediction. The results showed that among the three types of captured images (the natural state, artificially spread, and with the main axis removed), the detection accuracy for the images with the main axis removed was 99.38% (Wu et al., 2019). Deng et al. integrated the feature pyramid network into the Faster R-CNN network and proposed a rice grain per panicle measurement model for automatic recognition and counting of grains on the main branches of rice panicles, the overall recognition accuracy of the model was 99.4% (Deng et al., 2021).Gong et al. designed a full convolutional network based on U-Net and combined it with labeling for counting grains per panicle in rice, which has an error rate within 5 percent (Gong and Fan, 2022).

Due to the disadvantages of manual counting of rice panicles such as inefficiency, time-consuming and labor-intensive, research on software for convenient and fast counting is an inevitable trend. There are many researchers who have landed and transformed the technology into convenient and fast counting software. For instant, the P-TRAP software developed by Al-Tam et al. can analyze rice panicle structure and grain traits, as well as the count of per panicle (Al-Tam et al., 2013). Additionally, the PASTAR and PASTA, developed by Ikeda et al. can automatically extract values for length, number of branches, and number of grains from scanned panicle images (Ikeda et al., 2010). Deng et al. used the Cascade R-CNN method embedded in a feature pyramid network to establish a whole panicle grain detection (WPGD) model, and used the WPGD model to develop a system for automatic counting of per panicle in rice (Deng et al., 2022). Ma designed a hardware platform called the ‘grain-panicle integrated seed examiner,’ which uses a tablet computer instead of an industrial camera and a host PC. This platform can simultaneously measure the number of grains per panicle and the plant traits (Ma, 2018). However, in the study mentioned above, the mutual shading of rice panicles still affected the accuracy of the counting results to some extent.

In this study, we used smartphones to take images of rice panicles, while the CSRNet counting model (Li et al., 2018) based on deep learning is improved and applied to the problem of counting the number of grains per panicle in rice, and use curve fitting to correct the prediction results for eliminating the effect caused by shelter. On this basis, we developed the rice grain counting APP for Android and RiceGrainCounter, a batch counting software based on PC, to realize the rapid counting of rice grains per panicle.

## 2 Materials and methods

### 2.1 Materials

#### 2.1.1 Image capture of rice panicles

The rice panicles in this study were mainly from Zhangping City, Zhongshan, Putian City, and Xiapmen, Fuding City, China. Rice panicles were boxed and brought to the laboratory for photographs after being picked. The aim of this study was to detect the number of grains per panicle quickly and lossless, so no rice variety was specified. In order to verify the influence of light, camera equipment, distance and image size on the results, different phones were used in this study and do not set specific light source. A total of 4000 rice panicle images were collected in this study.

**Figure 1:**
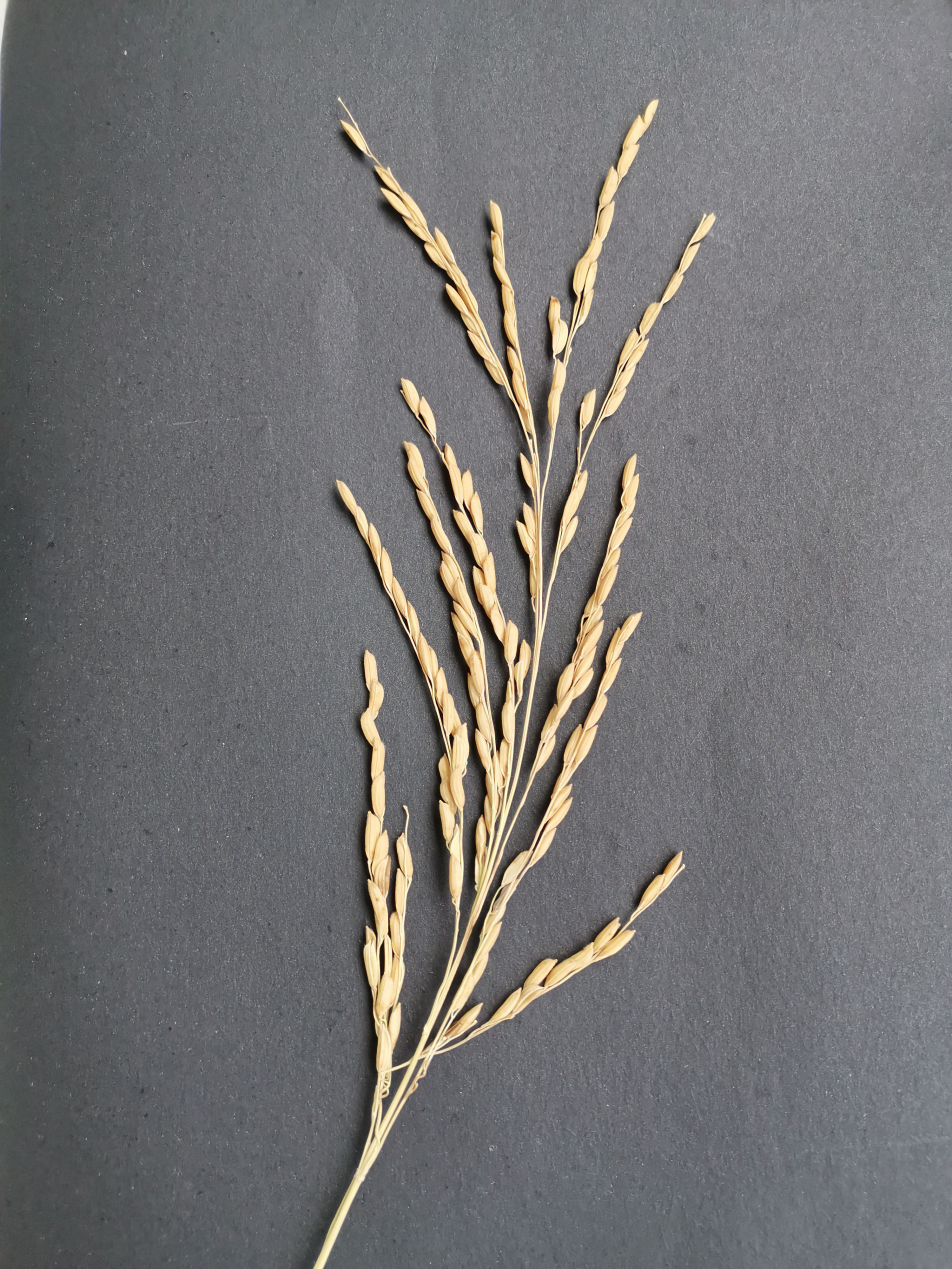
Rice panicle image. This illustration presents a rice panicle set against a black background, viewed from a top-down angle. It effectively highlights the overall structure of the rice panicle.

In this work, images of rice panicles were taken using the smartphone’s built-in camera. For photographing, the rice panicle samples were placed on black background paper, and the camera device was positioned 20-30 cm above the samples, parallel to the horizontal plane. The images of the rice panicle samples were then stored in.jpg format. In order to avoid incomplete capture due to shading, which could affect the final count of the panicle grains, the entire rice panicle was completely spread -out during shooting, and the individual branch stalks were kept from touching each other as much as possible (as shown in **Figure 1**). The shooting equipment parameters and image parameters are shown in **Table 1**.

**Table 1:** Shooting equipment parameters and image parameters.

Mobile phone brand	Camera model	Resolution	Number of images
HUAWEI	PCT-AL10	3000×4000	246
HUAWEI	SPN-AL00	1840×4000	674
HUAWEI	TAS-AL00	3648×2736	257
HUAWEI	COL-AL10	3456×4608	219
Vivo	iQ00 Neo 855	4032×3024	676
Vivo	Vivo x60t Prot	3060×4080	228
Realme	Realme X50 Pro 5G	4608×3456	564
Apple	iPhone 13	3024×4032	369
Apple	iPhone 13 pro	3024×4032	524
HONOR	FNE-AN00	3072×4096	243
Total			4000

#### 2.1.2 Image normalization processing

Since the images are taken with different phones, the image quality varies, so to accelerate the convergence speed of network training, the images need to be normalized before model training. Image normalization refers to a series of standard processing transformations of an image, so that it is transformed into an image with a fixed standard form, the standard image is called a normalized image.

Many researchers used the mean (mean = [0.485,0.456,0.406]) and standard deviation (std = [0.229,0.224,0.225]) from the ImageNet dataset to normalize the image. However, unlike the Image Net dataset which has a single image background and target object in this paper, the direct use of the mean and standard deviation of the Image Net dataset may have a strong bias. Therefore, in this paper, the algorithm proposed by Zou (Zou, 2022) is used to normalize the image, and the mean and standard deviation of the rice panicle image are obtained as follows: mean= [0.301, 0.294, 0.274], std= [0.189, 0.182, 0.163].

### 2.2 Methods

**Figure 2:**
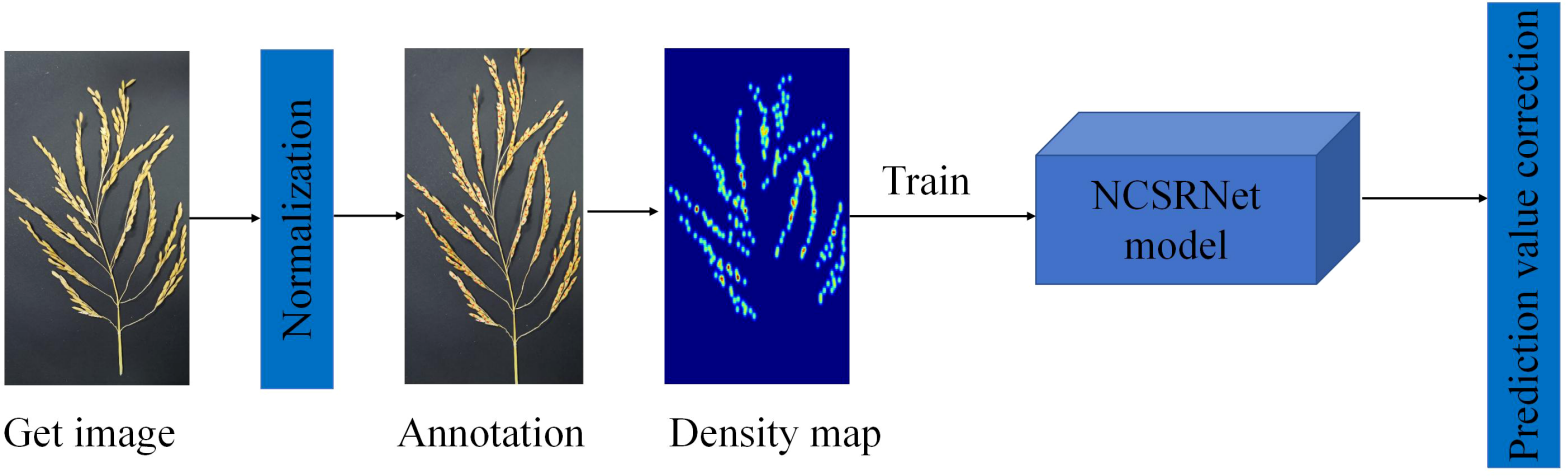
The flow chart of technical procedure. The diagram outlines the sequential steps in the technical process, which encompass image acquisition, normalization, annotation, density map generation, NCSRNet model training, and prediction correction.

In this study, the NCSRNet model was proposed by improving the CSRNet network model and applied to the study of grain count per panicle in rice, and the specific technology roadmap is shown in **Figure 2**.

#### 2.2.1 Rice panicle density map

(1) Image annotation

**Figure 3:**
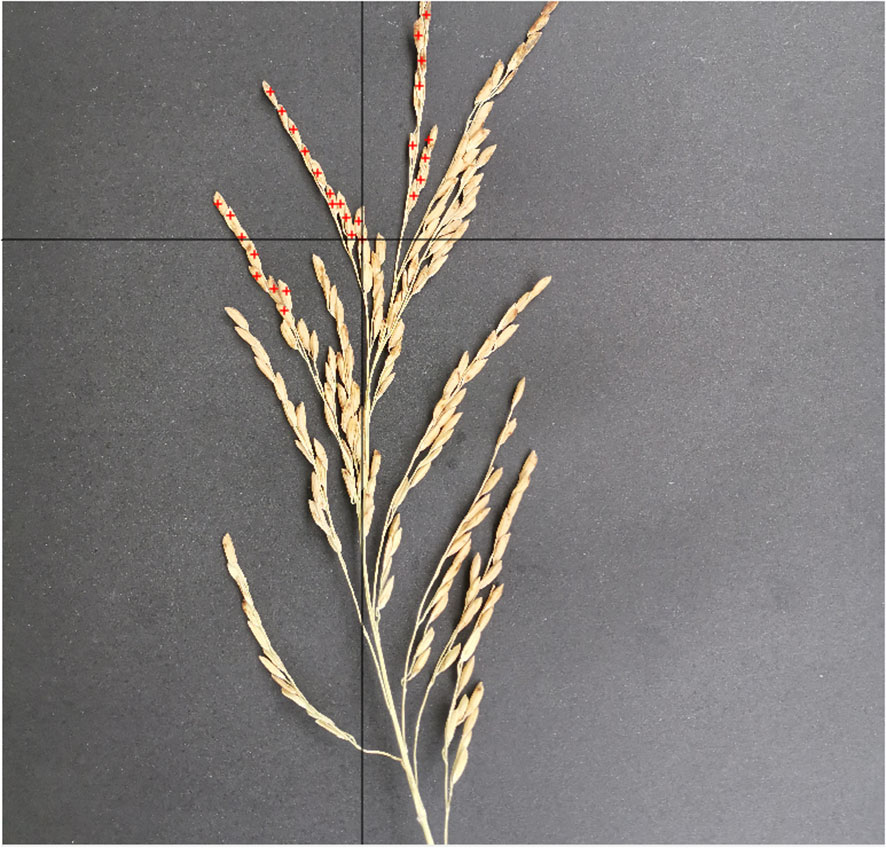
Marked process of rice panicle image. Red cross markers denote the annotated points on the rice panicle image, with a corresponding list on the right side detailing the coordinates of these specific points.

To facilitate the application of this study, we developed a concise image annotation software by ourselves, which is named as PointMarker. The image annotated by PointMarker, the position of the annotated panicles will be changed with the change of the picture when scaling, which makes it possible to make different sizes of the picture not to have to be re-labeled after the uniform resolution. There are three situations that need to be considered when annotating the position of the panicles. First, when the panicle is not covered, the annotation position is located in the center of the panicle as much as possible; second, when the panicle is partially covered, the annotation can be placed in the area where it is not covered; third, as the panicle is severely covered, even the human eye cannot be determined, the panicle will not be annotated. The annotation process is shown in **Figure 3**. The information about the location of the annotated panicles is stored in an Excel document, and the annotated file is saved in the form of.xlsx in the same path of the annotated image, with the same prefix name as that of the annotated image.

(2) Algorithm for generating density map of rice panicle images

The counting model based on convolutional neural network has two network input and output scheme configurations. The input of one is the image and the output is the number of targets in the image; the input of the other is the image and the output is the density map and the number of targets in the image is estimated from the density map. The second scheme was selected for this study, while the density map of each rice panicle image was generated using the method of Zhang et al. (Zhang et al., 2016). According to the generation method of the density map proposed by Zhang et al., the formula for calculating the density map is shown in Equation 1:


(1) $$F(x)=\sum_{i}^{N}\delta (x-x_{i})\;*\;G_{\sigma_{i}}(x),\;\sigma_{i}=\beta \bar{d^{i}}$$

Where, $\delta (x-x_{i})$ is an incremental function representing there is a panicle grain at pixel $x_{i}$ , $\sum_{i=1}^{N}\delta (x-x_{i})$ represents an image of a rice panicle marked with N grains. $G_{\sigma_{i}}(x)$ is a normalized Gaussian function which dynamically obtained extend parameter $\sigma_{i}$ by using an adaptive Gaussian kernel method. β is a hyperparameter. $\bar{d^{i}}$ represents the average distance from all neighbors to the rice grain at pixel $x_{i}$ . Assuming that the rice grain at pixel $x_{i}$ has k neighbors, the calculation formula is shown in Equation 2:


(2) $$\bar{d^{i}}=\frac{1}{k}\sum_{j=1}^{k}d_{j}^{i}$$

**Figure 4:**
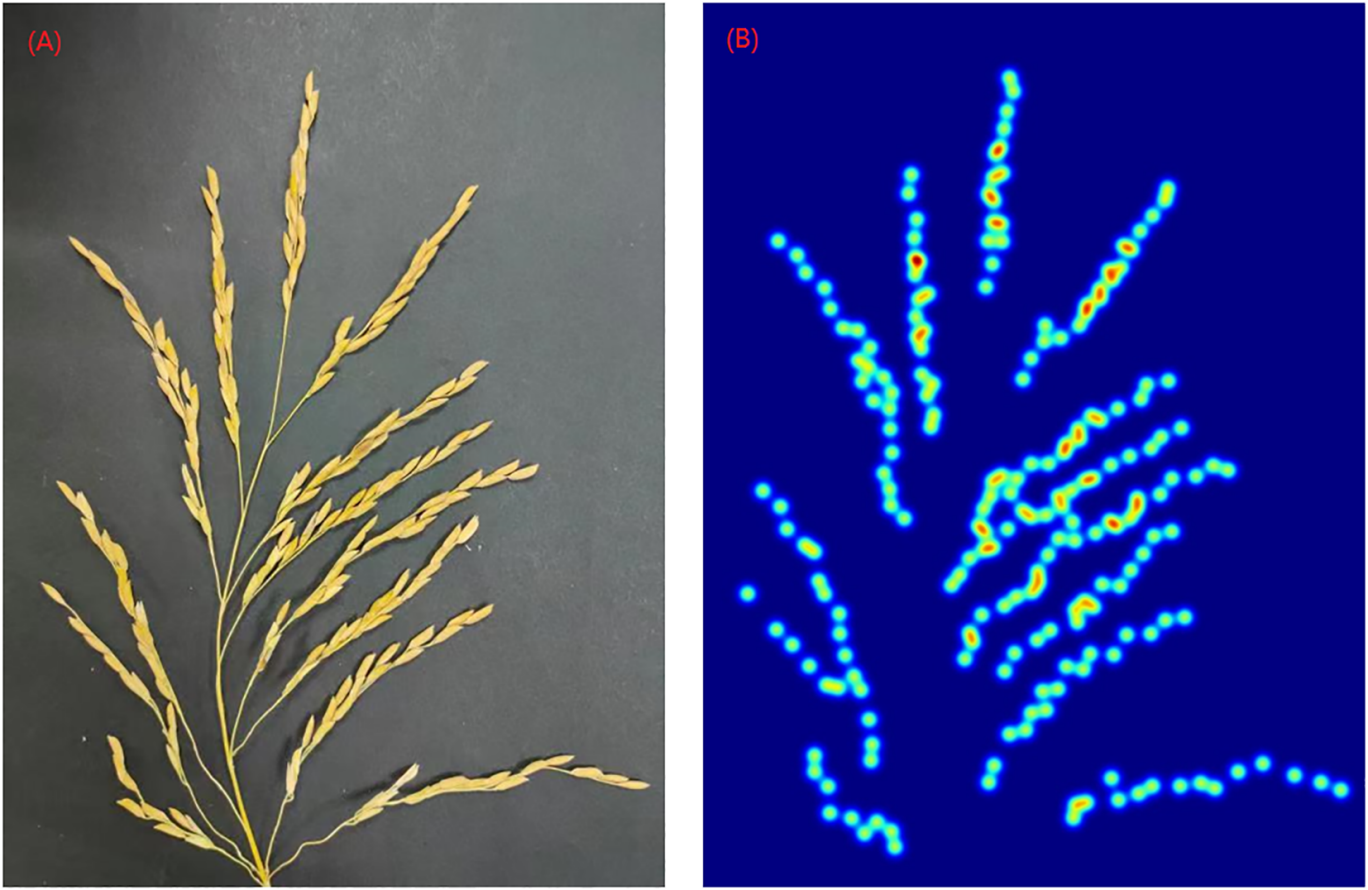
Rice panicle and its density map **(A)** Panicle of rice image **(B)** Density map of rice panicle.

In addition, the experimental results of Zhang et al. showed that β=0.3 gave the best results (Zhang et al., 2016), therefore, β=0.3 is used in this paper. **Figure 4** shows an example of the generated density map.

#### 2.2.2 NCSRNet model

**Figure 5:**
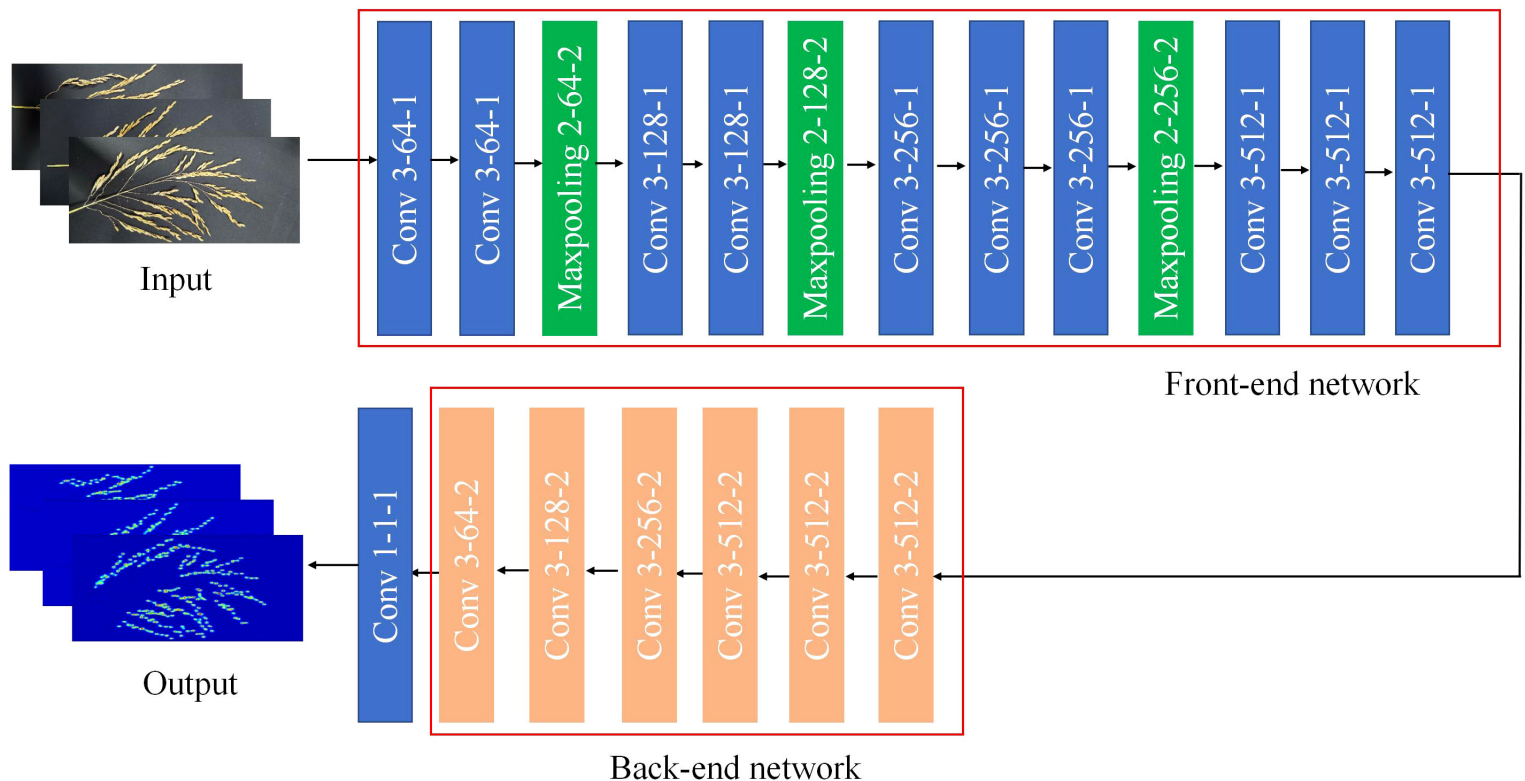
CSRNet model.

As shown in the **Figure 5**, the CSRNet model uses the first 10 layers of the VGG-16 network as the front end and the dilated convolution as the back end. This structure has the advantage of both utilizing the stronger migration learning capability of the VGG network to learn the feature information of the image, and using the dilated convolution to expand the receptive field without increasing the computational effort, maintaining the output resolution while extracting deeper saliency information.

(1) Loss function

The loss function measures the extent to which the predicted values differ from the actual data, and its generally used to measure how well the model predicts.

Three common loss functions are as follows:


(3) $$L(\theta )=\frac{1}{2N}\sum_{i=1}^{N}{\left\|Z(X_{i};\theta )-Z_{i}^{GT}\right\|}^{2}$$

(4) $$L_{C}(\theta )=\frac{1}{N}{\sum_{i=1}^{N}\left\|\frac{Z(X_{i};\theta )-Z_{i}^{GT}}{Z_{i}^{GT}}\right\|}^{2}$$

(5) $$L_{A}(\theta )=\frac{1}{N}\sum_{i=1}^{N}\left\|Z(X_{i};\theta )-Z_{i}^{GT}\right\|$$

In the above Equations 3–5, *N* is the number of images used to train the model, $X_{i}$ denotes the *i*th image in the training set, θ represents the parameters of the model, $Z(X_{i};\theta )$ is the total number of panicles in the density map predicted by the network, and $Z_{i}^{GT}$ indicates the true value of the number of panicles in the *i* th map. Equations 3–5 are the Euclidean loss function, relative loss function, and absolute loss function, respectively.

The distribution of grains in the rice panicle images in this study has problems such as overlapping and occlusion, uneven sparse and dense distribution areas, and differences in image scale. A single Euclidean loss can cause the model to overly focus on high value dense areas due to the square amplification effect, ignoring the subtle errors in sparse areas. After adding relative loss, the scale difference can be eliminated by calculating the ratio of error to true value, allowing the model to equally focus on different density regions. After adding absolute loss, it can reduce the interference of noise samples (such as occlusion and overlap) on model training and improve the model’s fault tolerance. Therefore, the loss function in this paper adds relative loss and absolute loss on the basis of the original Euclidean loss of CSRNet. The improved loss function is shown in Equation 6:


(6) $$L_{loss}=L(\theta )+\alpha L_{C}(\theta )+\beta L_{A}(\theta )$$

Where the α and β represent the balance factor, after a number of experiments and taking into account the evaluation indicators, it was determined that α=0.1 , β=0.01 .

(2) Model evaluation indicators

The model evaluation indicators are mainly used to assess the generalization ability of the model (i.e., the performance of a trained model in the validation set) and to optimize the model step-by-step. The evaluation indicators used in this paper are Mean Absolute Error (MAE), Mean Square Error (MSE), Root Mean Square Error (RMSE) and Mean Absolute Percentage Error (MAPE). The calculation formula is shown in Equations 7–10). The smaller the value of each evaluation indicator is, the better the model trained by the network is.


(7) $$MAE=\frac{\sum_{i=1}^{N}\left|C_{i}-C_{i}^{GT}\right|}{N}$$

(8) $$MSE=\frac{1}{N}\sum_{i=1}^{N}\left|{\left(C_{i}-C_{i}^{GT}\right)}^{2}\right|$$

(9) $$RMSE=\sqrt{\frac{1}{N}\sum_{i=1}^{N}{\left(C_{i}^{GT}-C_{i}\right)}^{2}}$$

(10) $$MAPE=\frac{1}{N}\sum_{i=1}^{N}\left|\frac{C_{i}-C_{i}^{GT}}{C_{i}^{GT}}\right|$$

In Equations 7–10, *N* denotes the number of images in the validation set, $C_{i}$ indicates the number of panicles in the *i*th image predicted by the model, and $C_{i}^{GT}$ represents the actual number of panicles in the *i*th image.

#### 2.2.3 Revision of predictions

**Figure 6:**
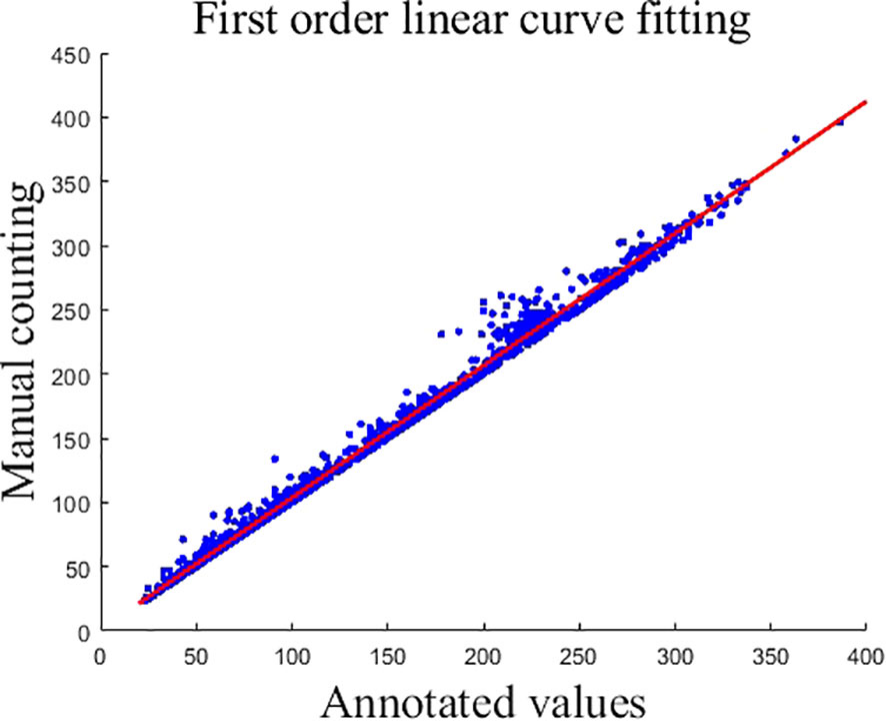
First order linear curve fitting. This graph represents the outcome of first-order linear curve fitting where the x-axis corresponds to the annotated values and the y-axis reflects manual counting results. Data points are indicated by blue dots, while the fitted linear curve is shown as a red line.


Due to the mutual shading of the panicles, which resulted in the existence of some panicles that were not labeled during manual labeling, causing the counts of the generated true density map to deviate from the original manual counts. To correct this error, the labeled counts and manual counts were fitted using a first-order linear curve, and the obtained first-order linear fitting curve is shown in Equation 11, and the fitted curve graph is shown in **Figure 6**.

(11) y=1.0294x+0.6069

In **Figure 6**, each blue circle represents the numerical information of an image, the x-coordinate of the blue dot is the labeled value, and the y-coordinate is the manually counted value.

The output is corrected using Equation 11. For data correction, x is the model output and y is the final output.

### 2.3 Experimental environment and dataset production

The network operates by configuring the Pytorch library with Anaconda. A Graphics Processing Unit (GPU) is used to perform accelerated computing. The specific experimental environment configuration is shown in **Table 2**.

**Table 2:** Experimental environment configuration.

Parameter	Value
Environment	PyTorch 1.10.0, Python 3.9, Cuda 11.3
GPU	RTX 3080 * 1
CPU	16-core Intel(R) Core(TM) i7-11700K @ 3.60GHz
Hard disk	System disk:256 GB, Data disk:2TB

The acquired 4000 images and their corresponding density maps are randomly divided into train set, test set and validation set in the ratio of 8:1:1. Then the number of images in the train set, test machine and validation set are 3200, 400 and 400 respectively. The model is trained and tested using the train set and test set respectively, and the validation set is used as the final test sample. The distribution of images taken by each model of cell phone is shown in **Table 3**.

**Table 3:** The detail of sample distribution.

Camera model	Train set	Test set	Validation set	Total
PCT-AL10	197	25	24	246
SPN-AL00	539	67	68	674
TAS-AL00	206	26	25	257
COL-AL10	175	22	22	219
iQ00 Neo 855	541	68	67	676
Vivo x60t Prot	183	23	22	228
realme X50 Pro 5G	451	56	57	564
iPhone 13	295	37	37	369
iPhone 13 pro	419	52	53	524
FNE-AN00	194	24	25	243
Total	3200	400	400	4000

### 2.4 Software development of NCSRNet model

**Figure 7:**
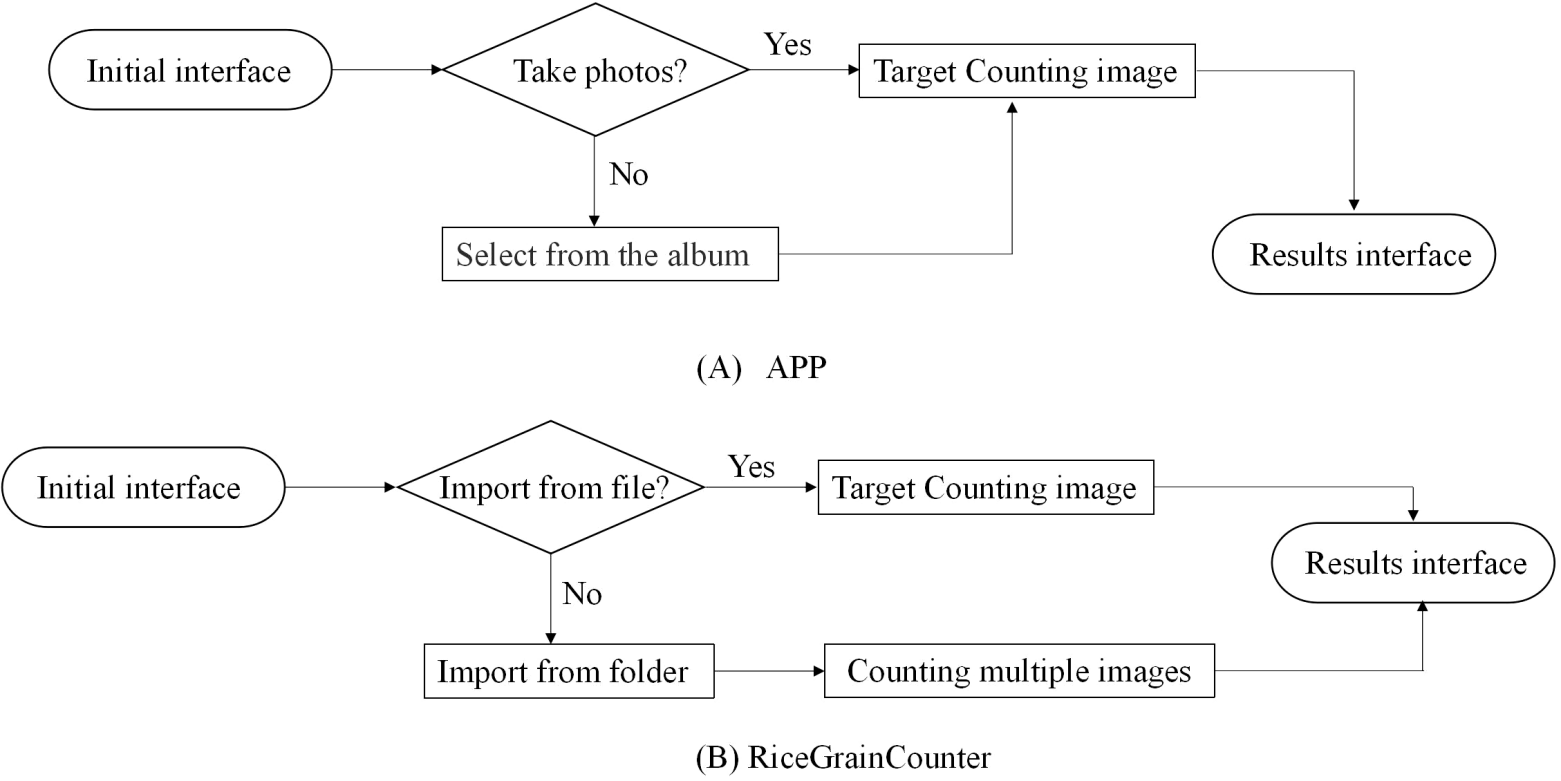
Flow chart of rice panicle number counting. **(A)** The counting flow of APP **(B)** The counting flow of RiceGrainCounter. Rice panicle counting is performed using an APP or the RiceGrainCounter software. With the APP, users can take photos or select images from their album and then view results. Using RiceGrainCounter, users can import files or select multiple images from a folder for counting before viewing results.

When the error rate of the NCSRNet model reaches the expected effect, the APP for counting of grain number per panicle based on Android and the batch counting software RiceGrainCounter based on PC are developed respectively. The APP can count one image at a time (the counting flow is shown in **Figure 7A**). RiceGrainCounter can count one image or multiple images (the counting flow is shown in **Figure 7B**).

## 3 Results

### 3.1 Behavior of the NCSRNet model during training

**Figure 8:**
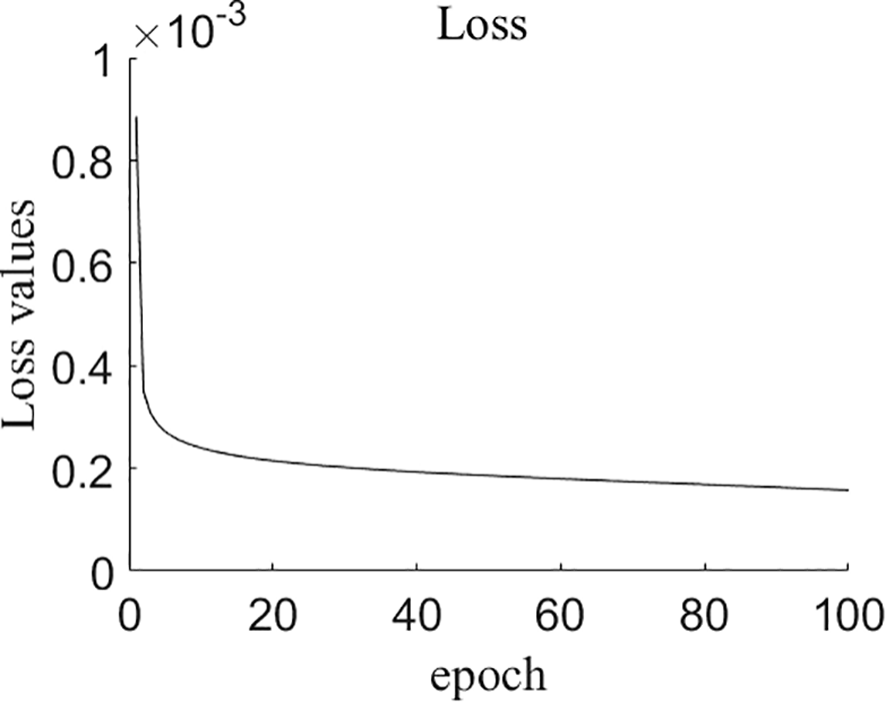
The changes of loss values during training process of NCSRNet model. As the number of epochs increases, the loss values gradually decrease to stabilize around approximately 0.00015.

**Figure 8** displays the change in the loss value of the model during the training process, which decreases as the number of training rounds increases. Subsequently, the decrease in the loss value becomes slower. After 80 epochs, the loss value remains stable in the range of 0.00015 to 0.00016. Therefore, training was terminated at 100th epoch.

### 3.2 Performance of the NCSRNet model

**Figure 9:**
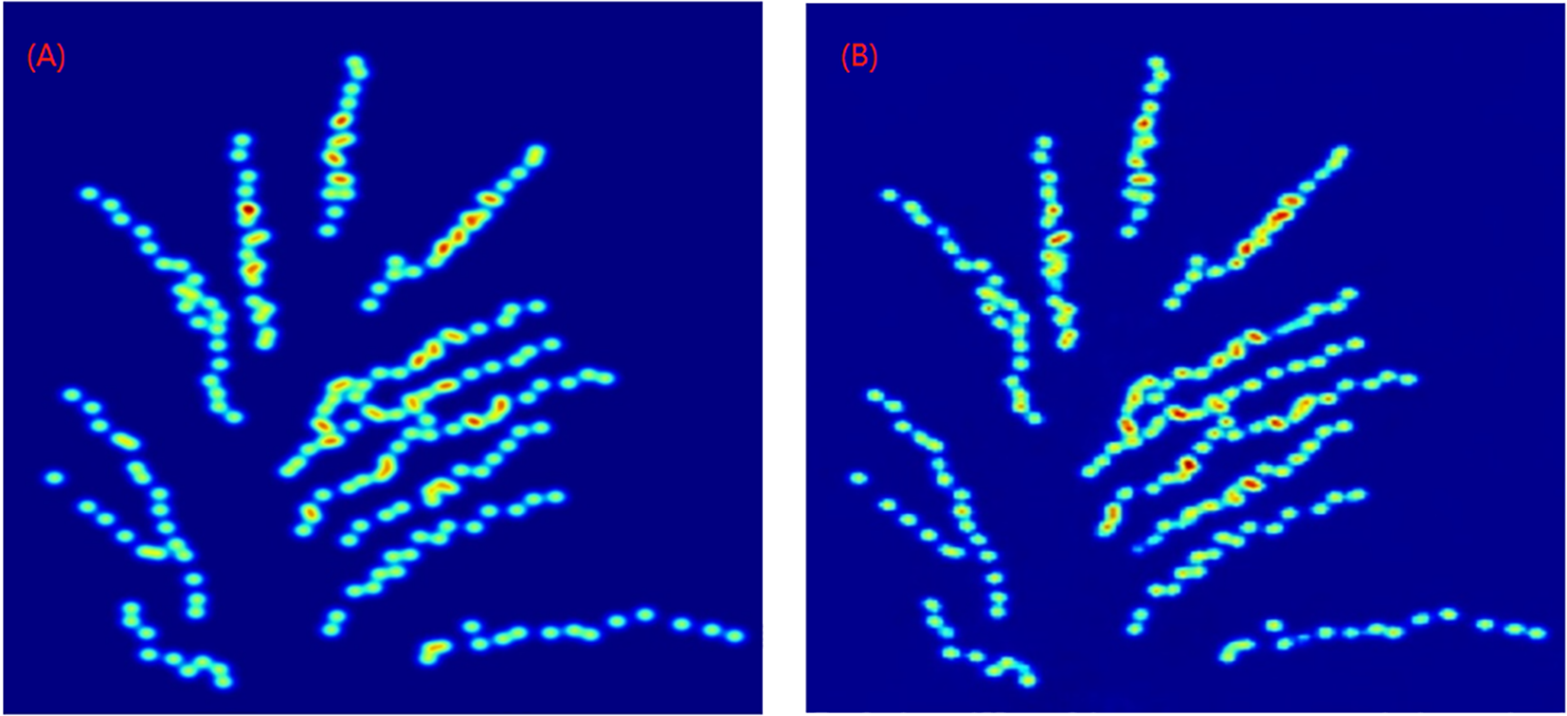
Detection results of the model **(A)** Truth density map **(B)** Generated density map.

The detection results of NCSRNet model are shown in **Figure 9**. As can be seen in **Figure 9**, the model is able to accurately identify and localize most of the panicles throughout the rice grain. Since the overlapping panicles in the major branches of the entire rice panicle image are small and often densely distributed, existing models of counting in grain number per panicle which based on deep learning are often unsatisfactory for identifying and detecting overlapping small grains. However, the model in this study was able to accurately detect most of the rice panicles under overlapping conditions.

### 3.3 Analysis of model assessment results

To further demonstrate the performance of the rice grain count per panicle model for detecting panicles on the entire panicle structure, we trained the CRSNet model using the same dataset. **Table 4** lists the values of the evaluation metrics of the two network models on the validation set.

**Table 4:** Values of evaluation index.

Model	MAE	MSE	RMSE	MAPE
CRSNet	5.8693	9.6304	2.4227	0.0435
NCRSNet	4.9659	7.8328	2.2284	0.0346

As can be seen from **Table 4**, the model in this study performs lower in the mean value of each evaluation indicator compared to the CRSNet model. This may be attributed to the fact that when training the network model, the sum of the Euclidean loss function, the relative loss function, and the absolute loss function were used as the loss function for model training in order to take into account the different sparsities among panicles. The identification and localization of heavily occluded panicles was enhanced by the inclusion of relative and absolute losses.

**Figure 10:**
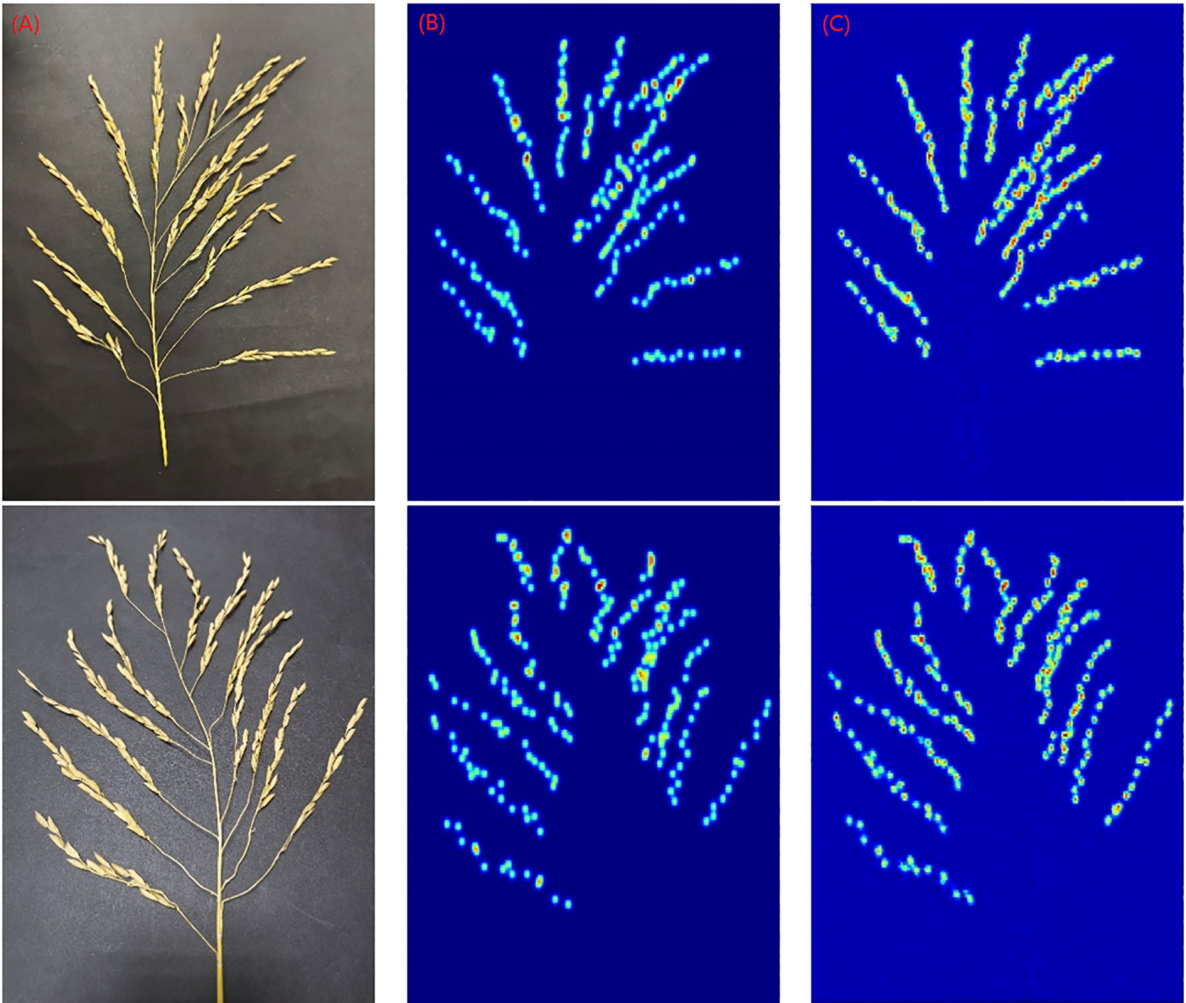
Detection results of the model **(A)** The origin image **(B)** Truth density map **(C)** Generated density map.

In order to more visually demonstrate the detection performance of the model, we used an additional 30 panicle images for a closer validation of the model. The 30 panicle images used are not part of the train set, test set, and validation set. Their prediction results are shown in **Table 5**. **Figure 10** shows the prediction results of 2 images taken out randomly.

**Table 5:** The further verified results of NCSRNet.

No.	Manual counting	NSCRNet forecast	Relative error
1	109	108	0.92%
2	199	191	4.02%
3	133	132	0.75%
4	205	208	1.46%
5	172	165	4.07%
6	276	268	2.90%
7	206	210	1.94%
8	140	145	3.57%
9	187	180	3.74%
10	169	173	2.37%
11	104	109	4.81%
12	187	193	3.21%
13	127	133	4.72%
14	165	154	6.67%
15	160	154	3.75%
16	250	229	8.40%
17	343	334	2.62%
18	226	215	4.87%
19	210	202	3.81%
20	186	181	2.69%
21	225	217	3.56%
22	164	154	6.10%
23	166	159	4.22%
24	98	104	6.12%
25	276	252	8.70%
26	183	171	6.56%
27	98	99	1.02%
28	220	215	2.27%
29	174	173	0.57%
30	113	118	4.42%
Mean error			3.83%

The data values in **Table 5** reveal that the largest relative error value is the image numbered 25, with an error value of 8.70%; the smallest error value is the image numbered 29, with an error value of 0.57%. From the results of this set of data, the purpose of accurate and non-destructive detection was basically achieved. But when the final output was corrected, the lack of consideration of varieties resulted in different degrees of sparsity between the panicles in the obtained images and an unbalanced number of image samples, leading to a situation in which the predicted values were more than the manually counted values (e.g., the data for the rice spikes numbered 4, 7, 8, and 10-13 as well as 24, 27, and 30 in **Table 4**).

### 3.4 Ablation experiments

To evaluate the contributions of individual components of the loss function within NCSRNet, we conducted ablation studies on the dataset. The loss function of NCSRNet is composed of three parts: Euclidean loss function, relative loss function and absolute loss function. Multiple loss variants were constructed to systematically analyze the impact of each loss function. The results are showed in **Table 6**. The baseline loss function only included Euclidean loss function. Variants were created by incorporating either the relative loss function or absolute loss function individually, followed by the loss function integrating both components. From **Table 6**, after by adding relative loss, the values of MAE, MSE, RMSE, and MAPE decreased from 5.8693 to 5.7614, from 9.6034 to 9.4171, from 2.4227 to 2.4003, and from 0.0435 to 0.0426, respectively, after by adding absolute loss, the values of MAE, MSE, RMSE, and MAPE decreased from 5.8693 to 5.7273, from 9.6034 to9.4568, from 2.4227 to 2.3932, and from 0.0435 to 0.0424, respectively. The complete loss function, which integrated both relative loss and absolute loss, achieved the minimum values of MAE, MSE, RMSE, and MAPE, the values were 4.9659, 7.8328, 2.2284, 0.0346, respectively. These results indicate that after adding relative loss and absolute loss, the values of the four evaluation indicators decreased significantly. These results also indicate the synergistic effect of collaborating with relative loss and absolute loss, which collectively enhance the model’s feature extraction.

**Table 6:** Ablation studies on each component of loss function.

No.	Components			MAE	MSE	RMSE	MAPE
	Euclidean loss	Relative loss	Absolute loss				
1	✓	×	×	5.8693	9.6304	2.4227	0.0435
2	✓	✓	×	5.7614	9.4171	2.4003	0.0426
3	✓	×	✓	5.7273	9.4568	2.3932	0.0424
4	✓	✓	✓	4.9659	7.8328	2.2284	0.0346

The ablation study shows that for the rice grain count per panicle problem, adding relative loss and absolute loss on the baseline loss function can improve the robustness and generalization of the model in different scenarios by focusing on learning samples with large prediction errors.

### 3.5 Software of rice grain count per panicle

#### 3.5.1 APP of rice grain count per panicle

**Figure 11:**
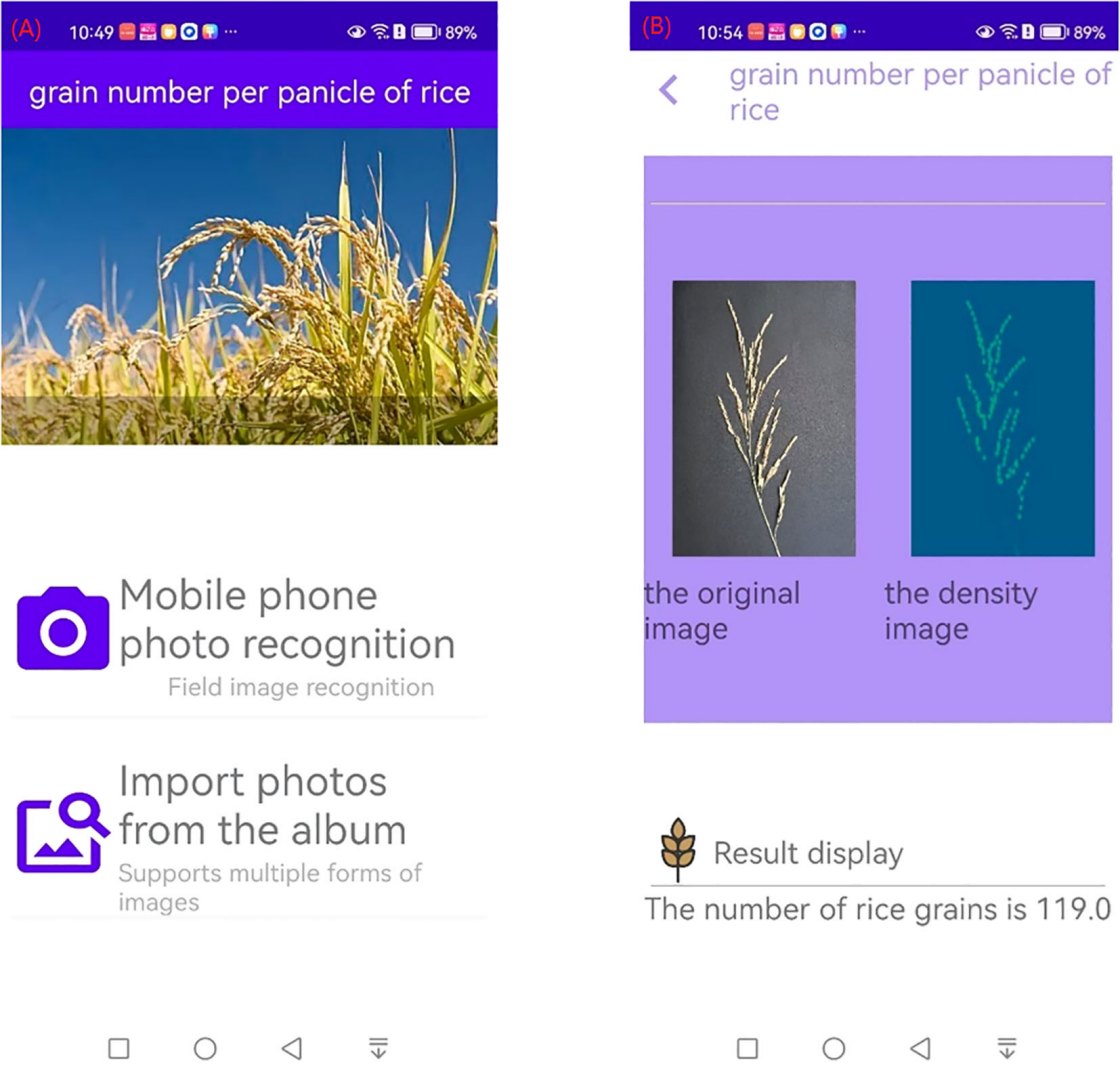
Interface of counting of grains per panicle in rice APP **(A)** Initial interface **(B)** Result interface.

The above model was further developed into an Android-based APP, which can take pictures of rice panicles from a cell phone album or by using the camera function of the cell phone, as shown in **Figure 11A**. The result interface is shown in **Figure 11B**, and the interface shows the original picture of the rice panicles, the density map, and the number of grains in the panicles.

#### 3.5.2 The batch software for counting grains per panicle in rice- RiceGrainCounter

**Figure 12:**
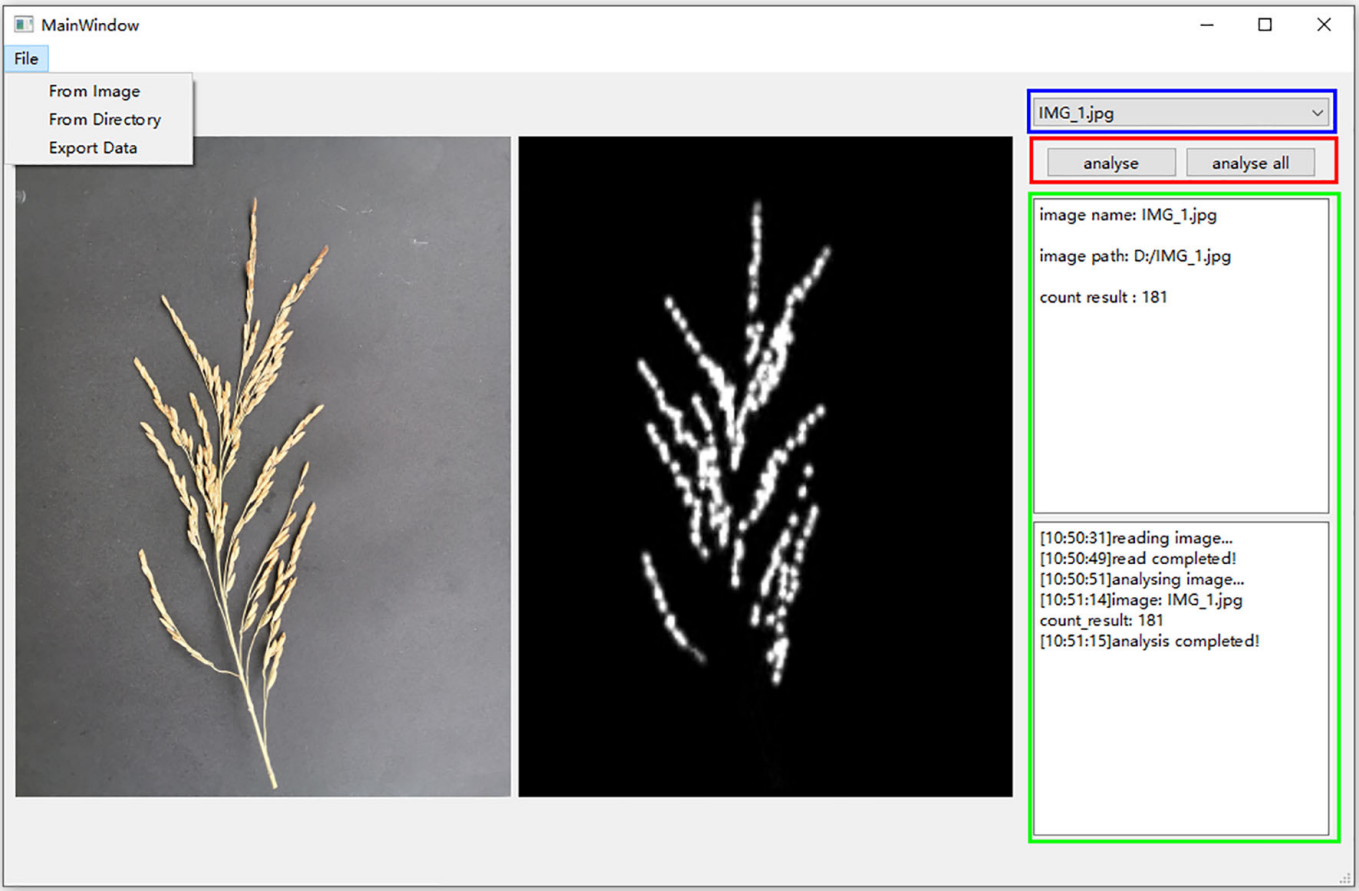
RiceGrainCounter main interface. On the left, the original image and its corresponding density map are displayed, while the right side shows the image name, path, and counting result.

The rice grain count per panicle APP facilitates rapid seed testing in the field, but it can only target one rice panicle at a time. In order to realize batch seed testing, this study developed RiceGrainCounter, a PC-based batch counting software, using a trained model. The software not only can count and analyze a single image, but also can analyze all the images in a folder in a batch. RiceGrainCounter’s File menu has three submenus, namely From Image, From Directory, and Export Data. The functions of From Image are to import an image to be analyzed, From Directory is to import images in a file to be analyzed, and Export Data is to export the analyzed data. The main interface of the software is shown in **Figure 12**. Below the File menu in the main interface are shown the currently analyzed images and their corresponding density maps, and the two buttons “analyse” and “analyse all” in the red box on the right are used to analyze a single image and all the images in a folder, respectively. The drop-down box in the blue box is used to view the analyzed images. The green frame displays information such as the name of the image, the number of grains detected, and the process of analysis. It is experimentally verified that the software completes the detection of one spike in 10 seconds on average, which basically meets the requirements of batch processing.

## 4 Discussion

This study focuses on solving the rice grain counting per panicle problem. In choosing the research method, this study introduces the CRSNet model used for crowd counting problem into the rice grain counting per panicle problem, and improves the original loss function of the CRSNet model, as well as corrects the data for the predicted results. The results show that the model trained in this study can accurately identify and localize the rice panicles in the image, and obtain precise counting results. In addition, we have also developed an APP for rice grain counting per panicle based on Android and RiceGrainCounter software based on PC on the basis of the above work.

The improved CSRNet model is not compared with other state-of-the-art methods in this paper. There are some reasons to consider: first, in the research of rice grain count pre panicle problem, most studies currently use object detection models (including one-stage detection algorithms such as YOLO (Sun et al., 2024), and two-stage detection algorithms such as Faster R-CNN (Deng et al., 2021; Wu et al., 2019), Cascade R-CNN (Deng et al., 2022), Mask R-CNN (Kong and Chen, 2021), etc.). The object detection model takes the original image as input and outputs as bounding box or point coordinates. The improved CSRNet model takes density maps as input and directly regresses count values. There are essential differences in the input and output forms between the two methods, and direct comparison lacks fairness. Second, Due to the lack of a public dataset for rice panicle images, researchers had to build their own dataset. For dataset annotation, object detection requires bounding box/point annotation, while density maps require point annotation and Gaussian kernel generation. The difference in annotation forms leads to the inability to fairly reproduce the detection model, resulting in unreliable comparability of the results. In addition, although this study did not compare it with object detection methods, comparing it with object detection methods is our next research focus. If there is a public dataset released, further exploration will be conducted.

Despite the fact that this study has made some progress in the problem of grain counting per panicle in rice, the following problems still exist:

1. This study requires that rice panicles picked in the field be brought back to the laboratory or photographed in the field and then tested for counting, and does not realize the function of taking photos and counting directly on the field plants. The realization of taking photos and giving the number of grains per panicle directly on the rice panicles in the field is an important next step.
2. Different rice varieties have different inter-panicles sparseness, but this study did not differentiate between rice varieties when obtaining the materials, which resulted in larger errors for panicles that were heavily adhered and shaded, and in data corrections that would result in larger predicted values than manually counted values.
3. There are certain limitations when it comes to taking images. For example, the background must be black and the rice panicles need to be spread out in order to be photographed, which affects the speed of taking pictures and results in some of the panicles drying up because they are not photographed in time. Quick and efficient shooting methods are therefore still a problem that needs to be solved.
4. While using the rice grain counting per panicle APP and the RiceGrainCounter software for PC, users are required to spread out each branch stalk of the rice panicle as much as possible when taking pictures of the panicle, so as to expose all the grains in the panicle. However, this study still spreads the rice panicle manually, which is similar to most of the current studies and does not address how to spread the panicle quickly, so looking for a fast and effective spreading method is the next research direction. In addition, the counting error for very heavily shaded rice panicles is large, and finding an effective way to eliminate this error is also the next step.
5. In this work, only the number of grains counted per panicle of rice was investigated, and the length of the main axis of the panicle, as well as other characteristics such as the number of branching stalks, were not calculated. For the calculation of the length of the main axis, AL-Tam et al. utilized spikes to fix the ends of the main axis to determine the starting position of the main axis before taking photographs, which added extra workload to the users of the software and was not suitable for high-throughput measurements (Al-Tam et al., 2013). In this study, when rice panicles were spread out and photographed in their natural state, it was found that the spindles would be bent to varying degrees, and the determination of the starting point of the spindles was also a major problem. Quickly obtaining the starting point of the main axis of the rice panicle from the image and accurately calculating the length of the main axis of the rice panicle with different degrees of bending are the focus of the next stage of research.

Currently, most of the yield estimation of rice, wheat and maize is focused on the method of measuring the number of spikes per unit area, and most of them have achieved certain research results (Lu et al., 2017; Hasan et al., 2019; Bao et al., 2020; Yang et al., 2022; Yang, 2020), but the number of grains per panicle is also an important factor in determining the yield, and influences the choice of breeding. In this study, we mainly improved the loss function. This improvement enhances the generalization and robustness of the model. In future research, the model can be deployed on agricultural embedded devices (such as UAV and agricultural sensors) for real-time counting of rice panicles in fields. After collecting images of different growth stages of rice panicles (heading stage, grain filling stage, maturity stage), this model can be used to calculate the number of grains per panicle at each growth stage, providing data support for the intrinsic connections between rice growth stages. At the same time, it also provides technical support for specific issues related to grain counting in other grains (such as wheat, corn, etc.) in the future, achieving accurate and rapid prediction, and providing guidance for effective evaluation of breeding. In addition, it also provides a new approach for the detection, localization, and counting of dense objects. The next step of this study is to collect images of rice panicles from different varieties in various countries and study the accuracy of the model in counting grains in different varieties of rice panicles. Adjust the model based on the initial data to achieve accurate and rapid counting of rice grains in each variety.

## Data Availability

The original contributions presented in the study are included in the article/supplementary material. Further inquiries can be directed to the corresponding author.
